# *Paraoxonase* single nucleotide variants show associations with polycystic ovary syndrome: a meta-analysis

**DOI:** 10.1186/s12958-020-00665-1

**Published:** 2020-11-20

**Authors:** Anthicha Kunjantarachot, Noel Pabalan, Hamdi Jarjanazi, Denise Maria Christofolini, Erik Montagna, Caio Parente Barbosa, Bianca Bianco

**Affiliations:** 1 Chulabhorn International College of Medicine, Thammasat University, Khlong Luang District, Rangsit, Pathumthani Thailand; 2 Environmental Monitoring and Reporting Branch, Ontario Ministry of the Environment, Conservation and Parks, 125 Resources Road, Toronto, Ontario Canada; 3 Human Reproduction and Genetics Center, Department of Collective Health, Faculdade de Medicina do ABC, Santo André, SP Brazil

**Keywords:** *PON*, *Paraoxonase*, Single nucleotide variants, PCOS, Meta-analysis

## Abstract

**Background:**

Etiology of polycystic ovary syndrome (PCOS) is attributed to genetic and environmental factors. One environmental factor is oxidative stress. Paraoxonase 1 (PON1) is an antioxidant high-density lipoprotein-associated enzyme encoded by the *PON1* gene. The *PON1* gene has been implicated in the risk for PCOS, the influence of which appears to come from single nucleotide variants (SNVs) at multiple genetic loci. However, association study reports have been inconsistent which compels a meta-analysis to obtain more precise estimates.

**Methods:**

From 12 publications, extracted genotype data were used in two genetic procedures. First, linkage disequilibrium (LD) was used to group eight *PON* SNVs into three: LD1, LD2 and LD3. Second, frequencies of the variant (*var*), wild-type (*wt*) and heterozygous (*het*) genotypes were used for genetic modeling (allele-genotype for LD1 and standard for LD2 and LD3). Risk associations were expressed in terms of pooled odds ratios (ORs), 95% confidence intervals (CIs) and *P*^a^-values. Evidence was considered strong when significance was high (*P*^a^ < 0.0001) and heterogeneity absent (I^2^ = 0%). Pooled effects were subjected to modifier (power), subgroup (Asian/Caucasian), outlier, sensitivity and publication bias treatments. Multiple comparisons were Bonferroni-corrected.

**Results:**

This meta-analysis generated 11 significant outcomes, five in LD1, six in LD2 and none in LD3. All six LD2 outcomes did not survive the Bonferroni-correction but two of the five in LD1 did. These two core LD1 findings conferred greater odds of PCOS to the *var* allele in the highly significant (*P*^a^ < 0.0001) overall (OR 1.44, 95% CI 1.24–1.67) and Asian (OR 1.41, 95% CI 1.20–1.65) outcomes. Of these two core outcomes, the Asian effect was homogeneous (I^2^ = 0%) but not the overall (I^2^ = 29%).

**Conclusions:**

Of the eight *PON* SNVs examined, two (rs854560 and rs662) were associated with PCOS risk. These 1.4-fold increased risk effects rendered Asians susceptible to PCOS. High statistical power, high significance, zero to low-level heterogeneity, robustness and lack of bias in the core outcomes underpinned the strong evidence for association.

**Supplementary Information:**

The online version contains supplementary material available at 10.1186/s12958-020-00665-1.

## Introduction

Polycystic ovary syndrome (PCOS) is a multifactorial and polygenic disorder [1]. Genetic and environmental factors have a marked influence on the progression of PCOS [2]. Oxidative stress is an environmental factor that plays an important role in the pathogenesis of PCOS [3] and has become the focus of genetic association studies [4, 5] where single nucleotide variants (SNVs) in genes with anti-oxidant function, like *paraoxonase 1* (*PON1*), have been implicated [6]. The *PON1* gene, located on chromosome 7q21.3, is composed of eight introns and nine exons spanning 26 kb and is a member of a multi-gene cluster including *PON1*, *PON2* and *PON3* [7]. All three *PON* genes lie on the long arm of chromosome 7 (7q21–22). *PON1* lies near the centromere, while *PON2* is near the telomere with *PON3* in between them [8]. The *PON2* SNV, rs7493 (Ser311Cys), is a substitution of guanine to cytosine nucleotides that results in serine to cysteine amino acid substitutions at residue 311 [9]. Studies have also revealed other SNVs in the *PON* gene cluster [10, 11]. Several SNVs in *PON1* have been studied in regard to its enzyme activity and risk of diseases including PCOS [12]. Among them are five in the promoter region: (i) rs854572 (g.95325384C > G), (ii) rs705381 (g.95324637 T > C), (iii) rs705379 (g.95324583G > A), (iv) rs854571 (g.95325307 T > C) and (v) rs854573 (g.95325551C > T) and two in the coding region (exon 6 and exon 3, respectively): (i) rs662 (c.575A > G, Gln192Arg) and (ii) rs854560 (c.163 T > A, Leu55Met) [13, 14]. These *PON1* variants (*var*) have been reported to regulate *PON1* expression and affect circulating serum levels as well as catalytic activity [13, 15]. The rs705379 polymorphism partially regulates *PON1* expression [16] by modulating the binding site for Sp1transcription factor [13, 17] while the rs705381 lies in a potential NF-1 transcription factor binding site [13]. *PON1* polymorphisms have been investigated in a variety of pathophysiological conditions that range from metabolic syndrome, cardiovascular diseases and stroke to diabetes. These reports collectively enabled better understanding of the genetic (in terms of transcription factor binding capacity and gene expression level) and physiological pathways conferring the importance of SNVs in oxidative regulation related to the etiology of PCOS. However, associations of the *PON* SNVs with PCOS risk have been contradictory, which gives reason to perform a meta-analysis of all eligible studies. Since the four previous meta-analyses [18–21], new primary studies have emerged. We provide a more comprehensive analysis and arrive at a reliable conclusion by reevaluating the associations of the *PON* SNVs with PCOS risk. To this end, we apply a number of meta-analytical tools that yields fresh insight in the *PON*-PCOS associations.

## Materials and methods

### Selection of studies

We searched MEDLINE using PubMed, Science Direct and Google Scholar for association studies as of August 03, 2019. The terms used were “*PON*”, “*paraoxonase*”, “polymorphisms”, “polycystic ovary syndrome” and “PCOS” as medical subject heading and text, unrestricted by language and time span. References cited in the retrieved articles were screened manually for additional eligible studies. Inclusion criteria were: (i) case–control studies evaluating the association between *PON* variants and PCOS and (ii) genotype frequency data to calculate odds ratios (ORs) and 95% confidence intervals (CIs). Exclusion criteria were: (i) animal studies, (ii) reviews, case report or case series, expert opinion and (iii) unusable genotype data.

### Data extraction

Two investigators (AK and NP) independently extracted data and arrived at consensus. The following information was obtained from each publication: indications (yes/no) of whether each article was included in the four previous meta-analyses [18–21], first author’s name, published year, country of origin, ethnicity, diagnostic criteria, *PON* SNVs examined and article features needed to tally the Clark-Baudouin score. Table S1 shows the rs numbers (SNVs per study), values under cases and controls that include sample sizes and genotype frequencies as well as minor allele frequencies and *P*-values for the Hardy-Weinberg Equilibrium (HWE).

### Statistical power, HWE and data distribution

We used the G*Power program [22] to evaluate statistical power, where adequacy was set at ≥80% assuming an OR of 1.5 and a genotypic risk level of α = 0.05. Control frequencies from the HWE were calculated from https://ihg.gsf.de/cgi-bin/hw/hwa1.pl with a two-tailed *P* < 0.05 indicating deviations. Data distribution was assessed with the Shapiro-Wilk test [23].

### Quality assessment of the studies

Methodological quality of the included articles was assessed with the Clark-Baudouin scale [24], the scores of which range from 0 (worst) to 10 (best) where < 5, 5–7 and > 7 indicate low, moderate and high, respectively.

### Meta-analysis

#### Linkage disequilibrium (LD) and genetic modeling

We performed LD analysis and adopted a suitable genetic model before examining associations of the *PON* SNVs with risk of PCOS. Proximity of SNVs has been posited to cause observed phenotype associations [25] and could merit grouping. Rationale for SNV grouping lies in the concept that SNVs in high LD are assumed to have similar association outcomes. D′ is the metric for LD, in which a value of 1 indicated complete LD [26]. Eight *PON* SNVs were grouped into three (Table S1) based on D′ values of 0.97–1.00 as LD1 (rs854560 and rs662), LD2 (rs705379, rs7493 and rs854572) and LD3 (rs705381, rs854571, rs854572 and rs854573). Because multiple SNVs had different notations for each genotype, we notated variant and wild-type as *var* and *wt*, respectively. Uniformity of the minor allele frequency (all < 0.50) values across the studies in LD2 and LD3 merited use of standard genetic models: (i) recessive: *var*-*var* versus *het* + *wt*-*wt*, (ii) dominant: *var*-*var* + *het* versus *wt*-*wt* and (iii) codominant: *var* versus *wt* [27]. Because of non-uniformity of the minor allele frequencies for LD1, we compared the following: (i) *var* allele with *var*-*wt*/*wt*-*wt* genotypes, (ii) *wt* allele with *var*-*wt*/*wt*-*wt* genotypes and (iii) *wt*-*var* (heterozygous) genotype with homozygous *wt*-*wt* and *var*-*var* genotypes.

#### Data synthesis

Risk association assessments were confined to HWE-compliant studies [28]. Using raw genotype frequency data, study-specific risks (ORs) of PCOS were estimated and pooled ORs and 95% CIs were calculated by comparing the effects on the same baseline. Associations were considered significant at a two-tailed *P*^a^ < 0.05, which were Bonferroni-corrected. Two indicators that were used to assess the strength of evidence were high significance (*P*^a^ < 0.00001) and homogeneity or zero heterogeneity (I^2^ = 0%). Analysis models for absence and presence of heterogeneity were fixed-effects [29] and random-effects [30], respectively. Heterogeneity was addressed in the following manner: (i) estimated with the χ^2^-based Q test [31] where significance was set at *P*_HET_ < 0.10 [32], (ii) quantified with the I^2^ statistic, which measures the degree of variability between studies [33] and (iii) its sources examined with outlier treatment using the Galbraith plot [34]. Outlier treatment divided the outcomes into pre-outlier and post-outlier. Robustness of the pooled ORs was assessed with sensitivity analysis, which involved serial omission of the studies followed by re-analysis. Publication bias was assessed with two criteria: (i) statistical significance and (ii) ≥ 10 studies [35]. Normal or non-normal distribution of the operating data (ORs) warranted use of either Egger’s regression asymmetry test [36] or Begg-Mazumdar test of correlation [37]. Data were analyzed using Review Manager 5.3 (Cochrane Collaboration, Oxford, England), SIGMASTAT 2.03 and SIGMAPLOT 11.0 (Systat Software, San Jose, CA).

## Results

### Search outcomes

**Fig. 1:**
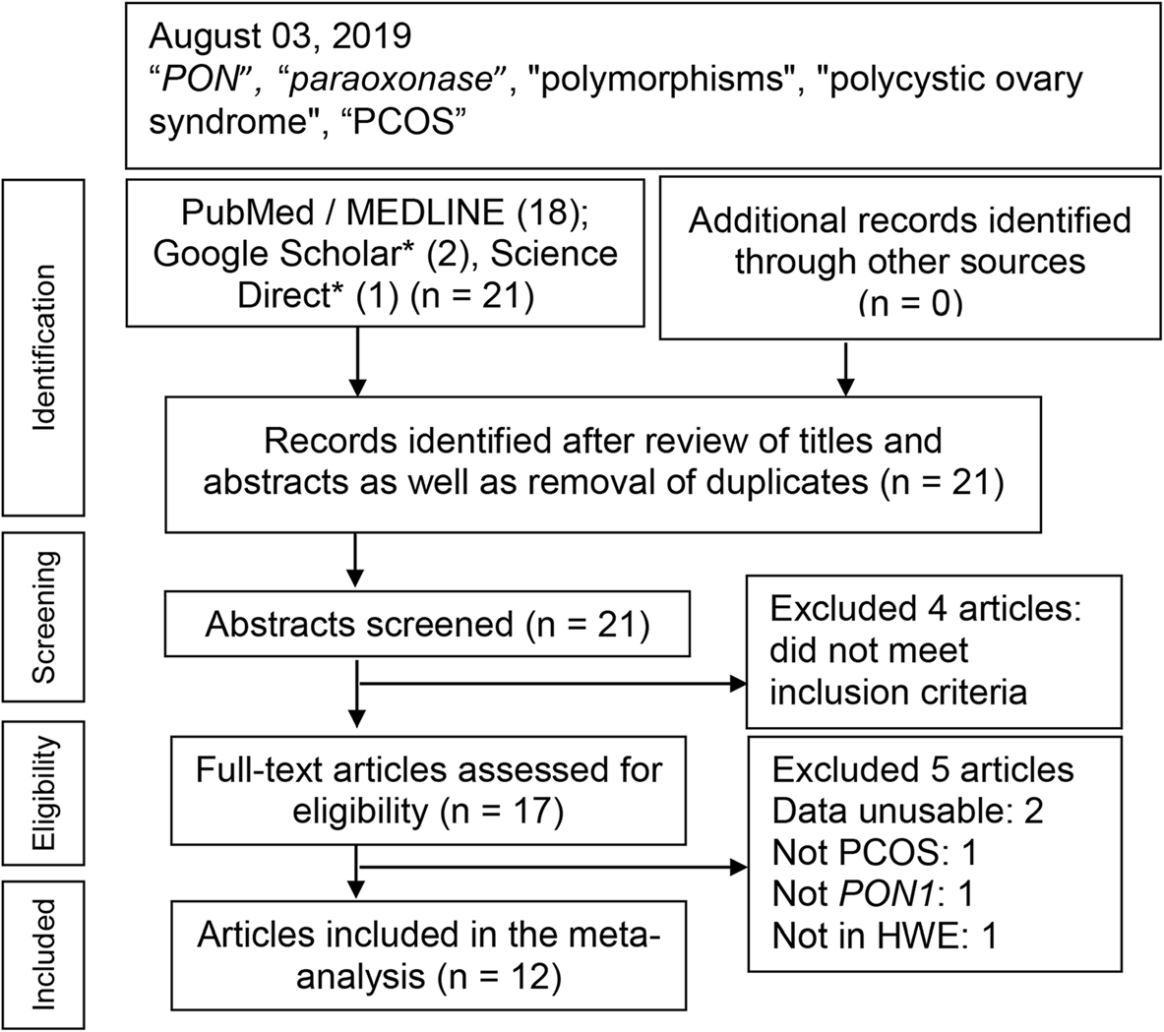
Summary flowchart of literature search. n: number of studies; * [all search terms in title] *PON*: *paraoxonase*; LD1: linkage disequilibrium 1; PCOS: polycystic ovary syndrome; HWE: Hardy-Weinberg Equilibrium

Figure 1 outlines the study selection process in a flowchart following the Preferred Reporting Items for Systematic Reviews and Meta-Analyses guidelines (Table S2). Twenty-one citations from the initial search were filtered to yield 12 articles for inclusion [38–49], all of which examined SNVs in *PON1* except Liu et al. [48], which focused on rs7493 SNV in *PON2*.

### Characteristics of the included studies

Table 1 shows which studies were and were not included in the four previous meta-analyses [18–21]. Three articles [47–49] were new additions to the meta-analysis literature and account for the updated associations between *PON* variants and PCOS. The sole African study [41] was included in the overall LD2 analysis, but not in LD1 (where it was HWE-deviating) nor in subgroup analysis. Asian and Caucasian subjects were in six and five articles of LD1, in five and three articles of LD2, respectively. Geography of the Asians was clearly dichotomous (China and India), which could have been sub-stratified were it not for issues of multiplicity (risk of false-positives) and reduced statistical power [50]. Based on the mean ± standard deviation (7.17 ± 1.19) of the normally distributed (Shapiro-Wilks test: *P* = 0.18) Clark-Baudouin scores, methodological quality of the component studies was high. Table S1 shows the unduplicated total sample sizes of the included articles (*n* = 7092), five [38, 39, 46–48] with adequate statistical power.

**Table 1:** Characteristics of the included studies that examined associations of *PON* single nucleotide variants with PCOS

	Included in previous meta-analyses				First author	[R]	Year	Country	Ethnicity	Diagnostic criteria	*PON1* variants	Clark-Baudouin score
	Liu [21]	Liao [20]	Gu [19]	Chen [18]								
1	yes	yes	no	yes	San Millan	[40]	2004	Spain	Caucasian	National Institute of Health	rs85460, rs662, rs705379	8
2	no	yes	no	no	Mohammed	[41]	2009	Egypt	African	National Institute of Health	rs854560^a^, rs705379	5
3	yes	yes	yes	yes	Lenarcik	[42]	2010	Poland	Caucasian	Rotterdam	rs854560	6
4	yes	yes	no	no	Zhang (*Ch*)	[43]	2011	China	Asian	Rotterdam	rs705379	6
5	yes	yes	yes	yes	Wang	[38]	2012	China	Asian	Rotterdam	rs854560, rs662	7
6	yes	yes	yes	yes	Paltoglu	[44]	2013	Greece	Caucasian	National Institute of Health	rs662, rs705379	8
7	yes	yes	yes	yes	Ferk	[45]	2014	Slovenia	Caucasian	Rotterdam	rs705379	6
8	yes	yes	yes	yes	Dadachanji	[46]	2015	India	Asian	Rotterdam	rs854560, rs662	8
9	yes	yes	yes	yes	Zhang	[39]	2015	China	Asian	Rotterdam	rs854560, rs662, rs705379	7
10	no	no	no	no	Dadachanji	[47]	2018	India	Asian	Rotterdam	rs705379, rs854571, rs854572, rs854573, rs705381	8
11	no	no	no	no	Liu	[48]	2019	China	Asian	Rotterdam	rs7493 (*PON2*)	9
12	no	no	no	no	Nalkiran	[49]	2019	Turkey	Caucasian	Rotterdam	rs854560, rs662^a^	8

*PON* *Paraoxonase*, *PCOS* polycystic ovary syndrome, *(Ch)* Chinese language, [R] reference number

^a^control frequencies deviated from the Hardy-Weinberg Equilibrium

### Meta-analysis outcomes

This meta-analysis generated 38 comparisons where 11 significant outcomes were confined to LD1 and LD2 (Tables 2 and 3), consigning *PON*-PCOS associations to five SNVs (rs854560, rs662, rs705379, rs7493 and rs854572).

**Table 2:** Overall and ethnic subgroup summary effects of the LD1 *PON* variants with PCOS

Comparison Genetic model number of cases/controls (pre-outlier)	*n*	Test of association			Test of heterogeneity			*n*	Test of association			Test of heterogeneity		
		OR	95% CI	*P*^a^	*P*_HET_	I^2^ (%)	Analysis model		OR	95% CI	*P*^a^	*P*_HET_	I^2^ (%)	Analysis model
	Pre-outlier							Post-outlier						
**Overall**														
2114/1588														
*var*	11	**1.44**	**1.24–1.67**	0.00001*†‡	0.17	29	Fixed	–	–	–	–	–	–	–
*wt*	11	0.81	0.57–1.17	0.26	0.00001	85	Random	10	0.90	0.84–1.10	0.59	0.16	31	Fixed
*het*	11	1.00	0.78–1.28	0.97	0.0001	74	Random	10	**0.85**	**0.76–0.96**	**0.008**†	0.60	0	Fixed
**Asian**														
1547/1270														
*var*	6	**1.41**	**1.20–1.65**	0.0001*†‡	0.99	0	Fixed	–	–	–	–	–	–	–
*wt*	6	0.94	0.74–1.20	0.62	0.03	60	Random	5	**0.83**	**0.80–0.98**	**0.03**	0.48	0	Fixed
*het*	6	**0.84**	**0.74–0.96**	**0.008**	0.42	0	Fixed	–	–	–	–	–	–	–
**Caucasian**														
567/318														
*var*	5	1.39	0.55–3.51	0.49	0.005	73	Random	4	1.01	0.63–1.61	0.97	0.36	6	Fixed
*wt*	5	0.67	0.24–1.84	0.44	0.0001	91	Random	4	1.09	0.77–1.53	0.63	1.00	0	Fixed
*het*	5	1.30	0.60–2.75	0.49	0.001	84	Random	4	0.91	0.65–1.29	0.61	0.52	0	Fixed

LD linkage disequilibrium, LD1 (rs854560 + rs662), *PON* *Paraoxonase*, *PCOS* polycystic ovary syndrome, *var* variant, *wt* wild-type, *het* heterozygous genotype (*var* + *wt*), *n* number of studies, OR odds ratio, CI confidence interval, *P*^a^ *P*-value for association, *P*_HET_ *P-*value for heterogeneity, I^2^ measure of variability; values in bold indicate significant associations; *survived the Bonferroni correction; † without evidence of publication bias; ‡ robust

**Table 3:** Overall, modifier and ethnic subgroup summary effects of the LD2 and LD3 *PON* variants with PCOS

SNV group Comparison Genetic model number of cases/controls (pre-outlier)	*n*	Test of association			Test of heterogeneity			*n*	Test of association			Test of heterogeneity		
		OR	95% CI	*P*^a^	*P*_HET_	I^2^ (%)	Analysis model		OR	95% CI	*P*^a^	*P*_HET_	I^2^ (%)	Analysis model
	Pre-outlier							Post-outlier						
**LD2**														
**Overall** (2006/1652)														
Recessive	9	1.33	0.93–1.88	0.11	0.0001	77	Random	6	**1.34**	**1.09–1.64**	**0.005**	0.21	30	Fixed
Dominant	9	1.09	0.90–1.31	0.37	0.02	56	Random	7	**1.16**	**1.02–1.33**	**0.03**	0.18	32	Fixed
Codominant	9	1.16	0.96–1.40	0.12	0.0001	80	Random	6	**1.14**	**1.03–1.26**	**0.01**	0.61	0	Fixed
**Power**^**a**^ (1448/1169)														
Recessive	3	**0.77**	**0.62–0.97**	**0.02**	0.15	48	Fixed	–	–	–	–	–	–	–
Dominant	3	0.93	0.81–1.06	0.27	0.12	52	Fixed	–	–	–	–	–	–	–
Codominant	3	0.90	0.74–1.09	0.28	0.04	70	Random	2	**0.82**	**0.72–0.93**	**0.002**	0.63	0	Fixed
**Asian** (1794/1484)														
Recessive	5	1.05	0.78–1.42	0.73	0.08	55	Random	3	1.22	0.97–1.55	0.09	0.81	0	Fixed
Dominant	5	1.04	0.91–1.18	0.59	0.39	1	Fixed	–	–	–	–	–	–	–
Codominant	5	1.03	0.93–1.13	0.59	0.12	49	Fixed	–	–	–	–	–	–	–
**Caucasian** (118/108)														
Recessive	3	1.91	0.94–3.89	0.08	0.08	61	Random	2	1.39	0.89–2.18	0.15	0.52	0	Fixed
Dominant	3	1.25	0.87–1.78	0.22	0.84	0	Fixed	–	–	–	–	–	–	–
Codominant	3	**1.32**	**1.05–1.67**	**0.02**	0.46	0	Fixed	–	–	–	–	–	–	–
**LD3**														
**Overall** (516/424)														
Recessive	4	1.02	0.74–1.40	0.91	0.05	63	Random	3	1.2	0.95–1.51	0.12	0.59	0	Fixed
Dominant	4	0.94	0.82–1.07	0.32	0.44	0	Fixed	–	–	–	–	–	–	–
Codominant	4	0.97	0.84–1.12	0.66	0.08	56	Random	3	1.04	0.93–1.17	0.49	0.75	0	Fixed

SNV single nucleotide variant, LD linkage disequilibrium, LD2 (rs705379 + rs7493 + rs854572), LD3 (rs854571+ rs854572 + rs854573 + rs705381), *PON* *Paraoxonase*, *PCOS* polycystic ovary syndrome; ^a^ statistical power ≥ 80%; *n* number of studies, OR odds ratio, CI confidence interval, *P*^a^ *P*-value for association, *P*_HET_ *P-*value for heterogeneity, I^2^ measure of variability; values in bold indicate significant association

#### LD1 associations with PCOS

**Fig. 2:**
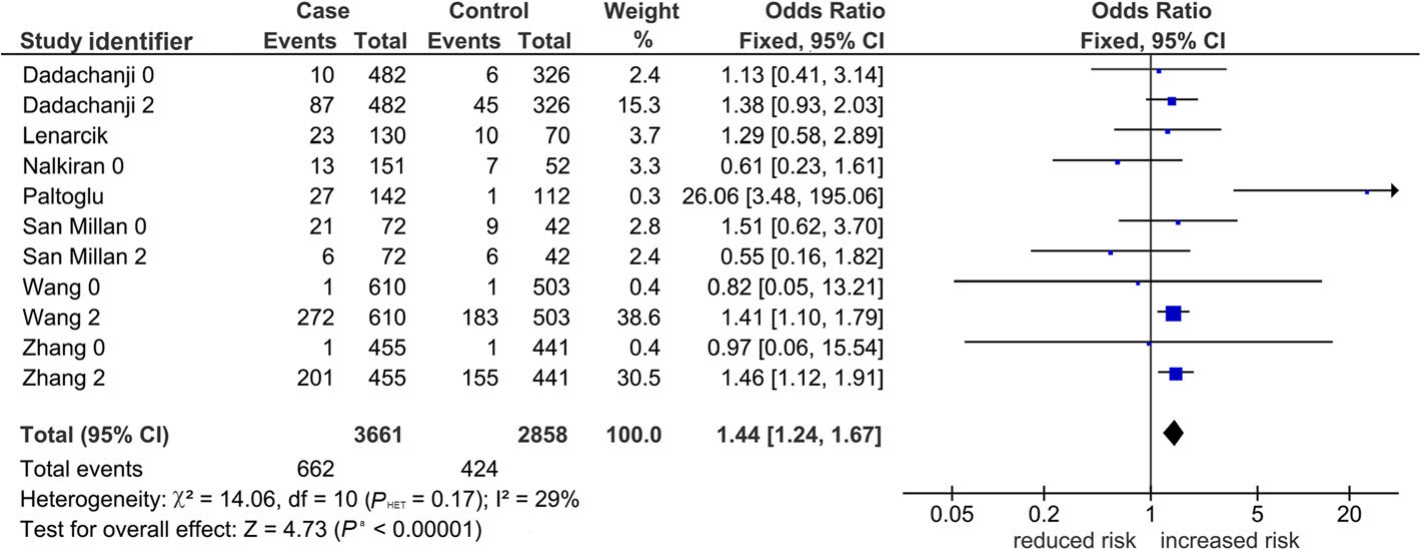
*PON* LD1 overall association with PCOS in the *var* model. The diamond indicates a pooled increased risk effect (OR 1.44), significant (*P*^a^ = 0.00001) with low-level heterogeneity (*P*_HET_ = 0.17, I^2^ = 29%). Squares indicate the OR in each study, with square sizes directly proportional to the weight contribution (%) of the study. Horizontal lines on either side of the squares represent 95% CIs. *PON*: *paraoxonase*; LD1: linkage disequilibrium 1; PCOS: polycystic ovary syndrome; *var*: variant; identifier after author name indicate 0 (rs854560) or 2 (rs662); OR: odds ratio; *P*^a^: *P*-value for association; *P*_HET_: *P-*value for heterogeneity; I^2^: measure of variability; CIs: confidence intervals

**Fig. 3:**
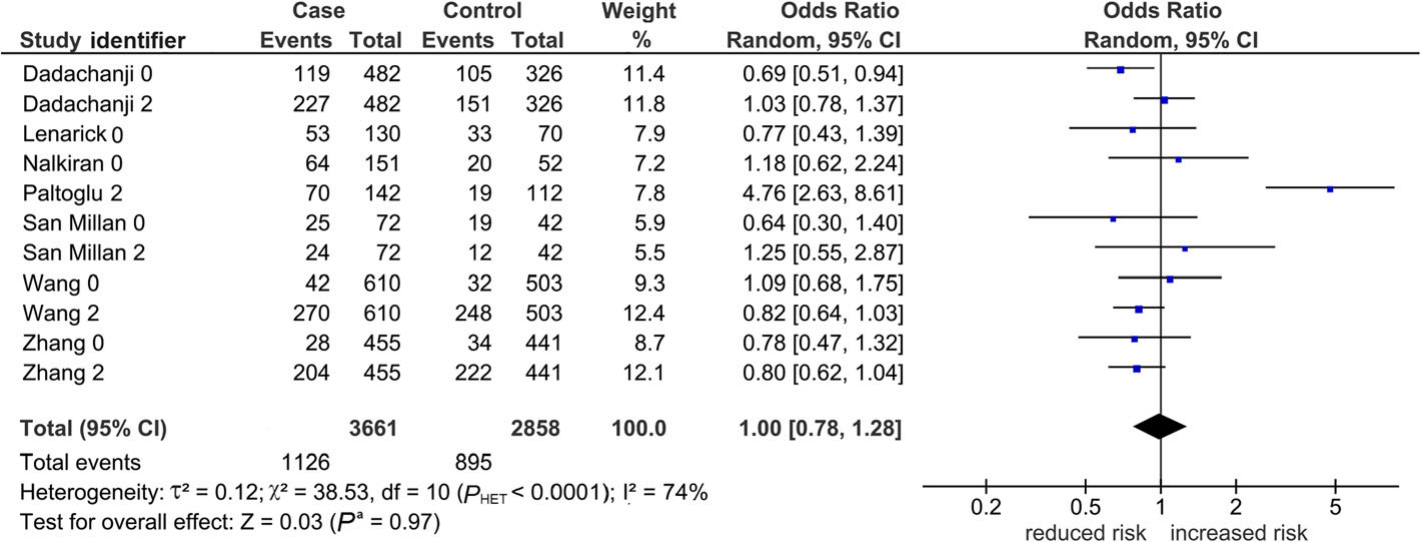
Pre-outlier association of *PON* LD1 with PCOS, the overall *het* analysis. The diamond indicates a pooled null effect (OR 1.00), not significant (*P*^a^ = 0.97) and heterogeneous (*P*_HET_ < 0.0001, I^2^ = 74%). Squares indicate the OR in each study, with square sizes directly proportional to the weight contribution (%) of the study. Horizontal lines on either side of the squares represent 95% CIs. *PON*: *paraoxonase*; LD1: linkage disequilibrium 1; PCOS: polycystic ovary syndrome; *het*: heterozygous genotype (*var* + *wt*); identifier after author name indicate 0 (rs854560) or 2 (rs662); OR: odds ratio; *P*^a^: *P*-value for association; *P*_HET_: *P-*value for heterogeneity; I^2^: measure of variability; CIs: confidence intervals

**Fig. 4:**
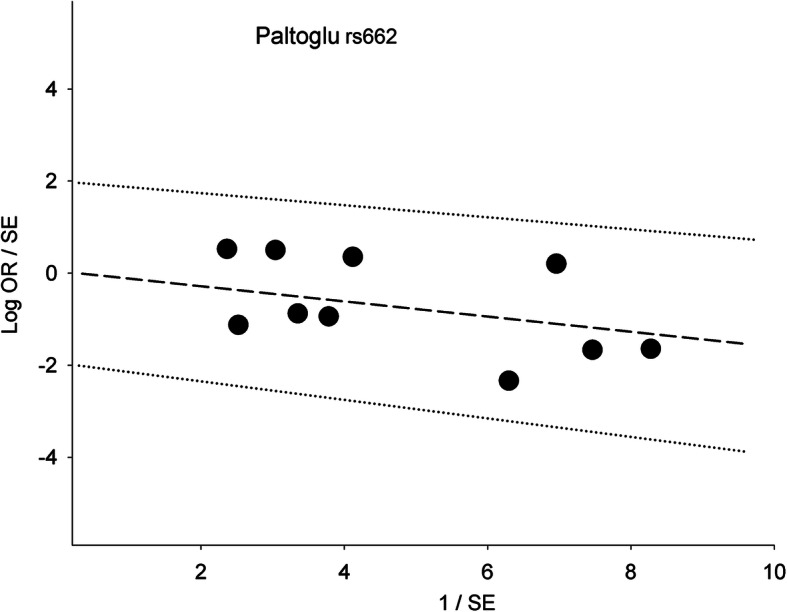
Galbraith plot analysis of *PON* LD1 with PCOS, the overall *het* analysis. The study found above the + 2 confidence limit was identified as the outlier. *PON*: *paraoxonase*; LD1: linkage disequilibrium 1; PCOS: polycystic ovary syndrome; *het*: heterozygous genotype (*var* + *wt*); OR: odds ratio; SE: standard error; Log OR: logarithm of odds ratio

**Fig. 5:**
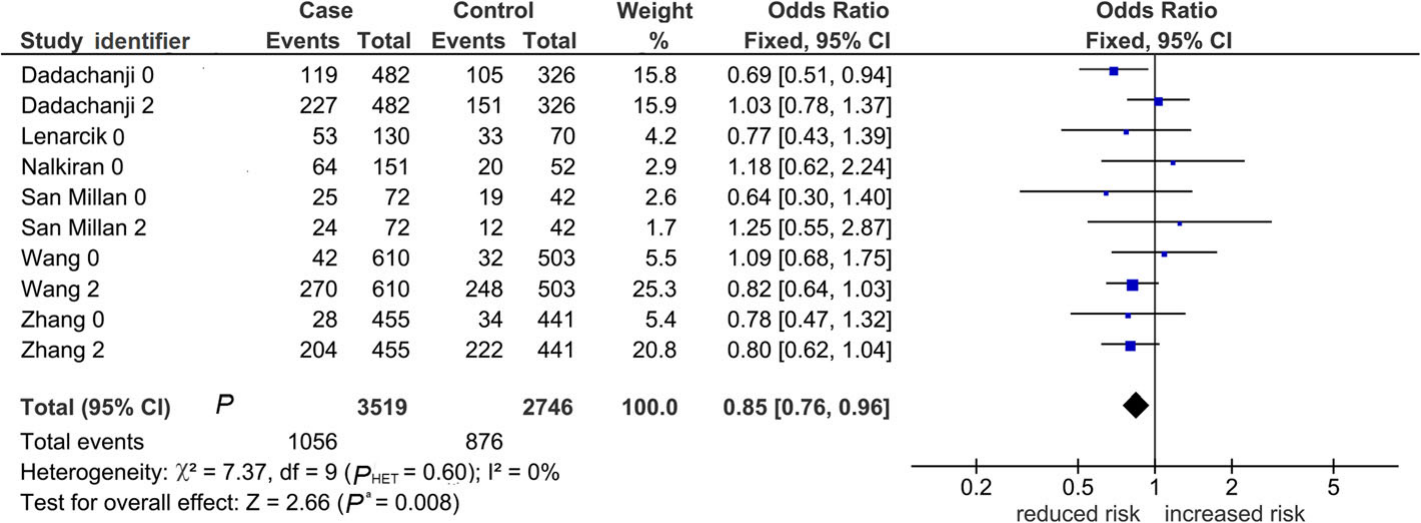
Post-outlier association of *PON* LD1 with PCOS, the overall *het* analysis. The diamond shows the pooled effect indicating decreased risk (OR 0.85), which was significant (*P*^a^ = 0.008) and homogeneous (*P*_HET_ = 0.60, I^2^ = 0%). Squares indicate the OR in each study, with square sizes directly proportional to the weight contribution (%) of the study. Horizontal lines on either side of the squares represent 95% CIs. *PON*: *paraoxonase*; LD1: linkage disequilibrium 1; PCOS: polycystic ovary syndrome; *het*: heterozygous genotype (*var* + *wt*); OR: identifier after author name indicate 0 (rs854560) or 2 (rs662); odds ratio; *P*^a^: *P*-value for association; *P*_HET_: *P-*value for heterogeneity; I^2^: measure of variability; CIs: confidence intervals

Table 2 shows 15 comparisons for LD1 (rs854560 and rs662), five of which were significant (*P*^a^ < 0.05). Of the five, two (*P*^a^ < 0.0001) survived the Bonferroni correction, found robust and both indicating increased risk in the *var* model. One was in the overall analysis seen in Fig. 2 with variable weight contributions of each study that ranged from 0.3% [44] to 38% [38], yet yielded a fixed-effects pooled effect (OR 1.44, 95% CI 1.24–1.67) with low-level heterogeneity (*P*_HET_ = 0.17, I^2^ = 29%). This pooled effect had no evidence of publication bias (Begg-Mazumdar test: *P* = 0.19). The other Bonferroni-surviving outcome was homogeneous (I^2^ = 0%), and found in Asians (OR 1.41, 95% CI 1.20–1.65). In contrast to the Asians, Caucasian outcomes were non-significant (*P*^a^ = 0.44–0.97). The mechanism of outlier treatment is visualized in the *het* LD1 overall comparison (Figs. 3, 4 and 5). In the pre-outlier forest plot (Fig. 3), the pooled OR was null (OR 1.00, 95% CI 0.78–1.28, *P*^a^ = 0.97) and heterogeneous (*P*_HET_ = 0.0001, I^2^ = 74%). Using the Galbraith plot, we identified the outlier [44] located above the + 2 confidence limit (Fig. 4). The post-outlier plot (Fig. 5) showed reduced risk (OR 0.85, 95% CI 0.76–0.96) with eliminated heterogeneity (*P*_HET_ = 0.60, I^2^ = 0%), acquired significance (*P*^a^ < 0.008), found robust and no evidence of publication bias (Eggers test: *P* = 0.53). However, this post-outlier pooled effect did not survive the Bonferroni correction.

#### LD2 and LD3 associations with PCOS

Table 3 shows 18 comparisons for LD2 (rs705379, rs7493 and rs854572), six of which were significant (*P*^a^ = 0.005–0.03) with none surviving the Bonferroni correction. Of the six, two indicated reduced risks in the recessive and codominant models, both in power analysis (ORs 0.77–0.82, 95% CIs 0.62–0.97, *P*^a^ = 0.002–0.02). In contrast, the remaining four pooled ORs indicated increased risks, three of which were post-outlier derived in the overall analysis (ORs 1.14–1.34, 95% CIs 1.02–1.64, *P*^a^ = 0.005–0.03) and one in the pre-outlier Caucasian subgroup of the codominant model (OR 1.32, 95% CI 1.05–1.67, *P*^a^ = 0.02). Table 3 shows five comparisons for LD3, none of which were significant (*P*^a^ > 0.05). Of the five, four had pooled ORs that skirted the null effect (ORs 0.94–1.02).

## Discussion

### Summary of findings

In the main findings, the two LD1 *var* outcomes met the criterion of high significance. However, only the Asian outcome met the other criterion of homogeneity. Nevertheless, these two core findings identified the *PON* rs854560 and rs662 SNVs to be associated with risk of PCOS. The LD groups differed by ethnicity, where a significant codominant risk effect in the LD2 was found in Caucasians (OR 1.32, *P*^a^ = 0.02) but not in Asians (OR 1.03, *P*^a^ = 0.59). In LD1, on the other hand, significant risk effects were found in all genetic models in Asians (*P*^a^ = 0.0001–0.03) but not in Caucasians (*P*^a^ = 0.44–0.97). Two notes regarding power outcomes (i) LD1 Asian increased risk effects were also powered and homogeneous in the *var* comparison; and (ii) LD2 power effects were protective (18–23%) in the recessive and codominant models. Reduced risk effects (15–16%) were also observed in the *het* outcomes of LD1 (overall and Asian), where the phenotypic difference (increased risk versus reduced risk) between *var* and *het* suggests a heterosis phenomenon. Heterosis occurs when subjects heterozygous for a specific genetic polymorphism show a different phenotype from homozygotes [51], conferring a heterozygote advantage (protection). Variations of these effects between the genetic models and ethnic subgroups suggest complex *PON*-PCOS associations, which is further driven by interactions between genetic and non-genetic risk factors. Gene-gene and gene-environment interactions have been reported to have roles in the associations of *PON* variants with PCOS. All 12 included articles acknowledged the role of environment but haplotype analysis was addressed in only four (33%) of the component studies [38, 45, 47, 49]. In addition to *PON*, one article [48] examined SNVs in another gene (superoxide dismutase-2).

The role of *PON1* variants with PCOS have been addressed in four meta-analyses [18–21], which we compared with the present study in terms of general features and methodology (Table S3). The previous meta-analyses [18–21] examined *PON1* only but our study included *PON2* (rs7493) on account of its full LD with rs705379 and rs854572 of *PON1*. To our knowledge, this is the fifth meta-analysis to address the *PON*-PCOS associations, but the first to accomplish the following: (i) perform an umbrella review (Table S3); (ii) apply outlier treatment with the most number (*n* = 12) of included articles; and (iii) operate within LD parameters. These features render our study as most comprehensive, managing to accomplish two things: (i) fill the gaps and update the meta-analysis knowledge of the *PON* SNV-PCOS associations and (ii) minimize the methodological problems that beset primary studies including limited statistical power, unrecognized confounding factors, misleading definition of phenotypes and stratification of populations [24].

### Physiological correlates

PCOS is a reproductive endocrinopathy [52] that is associated with dyslipidemia, obesity and insulin resistance [53]. These metabolic disorders lead to disease conditions such as hypertension, cardiovascular disease and diabetes mellitus [54]. These disease comorbidities were shown to be related with increased oxidative stress, exhibiting altered physiological conditions such as increased plasma glucose and low antioxidant reserves [55, 56]. Low antioxidant levels in PCOS patients suggest that elevated oxidative status contribute to the battery of cardiometabolic derangements [57]. These perturbations have been attributed to reduced serum PON1 activity in PCOS patients [58], its genetic underpinnings [59] partly explained with the significant findings of our study observed in LD1, involving rs854560 (L55M) and rs662 (Q192R). These two SNVs account for the main PCOS risk associations in this meta-analysis. The rs854560 variant, not rs662, has been reported to affect enzyme concentration [15, 60]. Compared to 55LL genotype in rs854560, 55MM carriers have lower enzyme activity, which may be attributed to the correlation between the 55 M allele and reduced mRNA and protein levels [61]. Moreover, strong LD of this variant with rs662 may partly explain the variation in PON1 catalytic activity [62]. Depending on the assay used, the R allele of rs662 could be associated with increased or decreased PON1 activity [63]. Reduced enzyme activity leads to elevated levels of oxidative stress altering the metabolism in PCOS patients [64]. Oxidative stress profiles in these patients revealed the R allele carriers had impaired physiologic responses involving increased oxidization of low-density lipoprotein [46], high triglyceride levels [38] and elevated insulin resistance [44]. Even in the absence of insulin resistance, oxidative stress levels remain high in PCOS women [65]. This cascade of impaired physiological events, as well as inflammatory responses to cellular injuries caused by oxidative stress, primes the pathophysiology of PCOS as inflammatory mediators that have been known to regulate PON expression [66] and contribute to PCOS pathogenesis [67]. Moreover, genetic combination analysis demonstrated that haplotypes containing the 192R allele was significantly associated with PCOS risk ranging from 1.6 to 8-fold [38, 49]. In sum, the R allele of rs662 appears to be a genetic determinant of PCOS susceptibility in the female population [49]. These genetically susceptible women are the likely candidates who could benefit from the clinical application of our results. PCOS risks along with their comorbidities could be clinically reduced or delayed with modifications of environmental influences meant to reduce levels of oxidative stress.

### Strengths and limitations

Limitations of our study include: (i) six (50%) of the included primary 12 articles were underpowered. However, statistical power at the aggregate level was more than adequate and (ii) credible subgrouping was suggested at no more than two levels [50], which left other possible subgroups (e.g. diagnostic criteria) unexamined. On the other hand, strengths of this meta-analysis are: (i) the combination of more studies, larger sample sizes and multiple meta-analysis treatments raised the level of evidence presented in this study; (ii) restricting our analysis to HW-compliant studies minimized the risk of representation and methodological bias [28]; (iii) potency of outlier treatment is evidenced from the LD1 and LD2 analyses, where six (50%) and eight (67%) of the 12 combined post-outlier comparisons were acquired significance and eliminated heterogeneity (Tables 2 and 3); (iv) the overall methodological quality (determined by the Clark-Baudouin Scale) of the included articles was high; (iv) all genotyping used polymerase chain reaction followed by either restriction fragment length polymorphism (11/12 articles) or direct sequencing (1/12 articles) techniques, indicating low-level heterogeneity; (v) all tissue samples were from blood, indicating source homogeneity and (vi) umbrella review of previous meta-analyses enabled comparisons of methodological treatments and findings (Table S3). This provided insight into the evolving consolidation of knowledge into the association genetics of PCOS involving the *PON* SNVs.

## Conclusions

Bonferroni-corrected significance identified rs854560 and rs662 (LD1) as the *PON* variants associated with PCOS. Subgrouping delineated ethnic-specific effects rendering *var* carrier Asians susceptible. Future studies exploring other ethnic groups would substantiate conclusions on these *PON*-PCOS associations with sample sizes appropriate for detecting small genotypic risks.

## Supplementary Information

**Additional file 1:** **Table S1.** Quantitative features of the included *PON*-PCOS studies. **Table S2.** Preferred Reporting Items for Systematic Reviews and Meta-Analyses checklist. **Table S3.** Comparisons between meta-analyses that examined the *PON* variants associations with PCOS.

## Data Availability

All data generated or analyzed during this study are included in the supplementary information files.
