# Supplementary material for: Paraoxonase single nucleotide variants show associations with polycystic ovary syndrome: a meta-analysis
Source: Reprod Biol Endocrinol. 2020 Nov 20;18:114. doi: 10.1186/s12958-020-00665-1 (PMC7678182; doi:10.1186/s12958-020-00665-1)
Supplement: Supplementary file 1 — Additional file 1: Table S1. Quantitative features of the included PON-PCOS studies. Table S2. Preferred Reporting Items for Systematic Reviews and Meta-Analyses checklist. Table S3. Comparisons between meta-analyses that examined the PON variants associations with PCOS. [file 12958_2020_665_MOESM1_ESM.pdf]

Table S1 Quantitative features of the included *PON*-PCOS studies

					Sample sizes				Case			Control				
	Author Group	Year	Ethnicity	rs number	Case	Control	Total	Power † (%)	*wt-wt*	*wt-var*	*var-var*	*wt-wt*	*wt-var*	*var-var*	minor allele frequency	HWE
	**LD1**															
1	Lenarcik	2010	Caucasian	rs854560	130	70	200	27	54	53	23	27	33	10	0.379	0.987
2	Nalkiran	2019	Caucasian	rs854560	151	52	203	24	74	64	13	25	20	7	0.327	0.363
3	San Millan	2004	Caucasian	rs854560	72	42	114	18	26	25	21	14	19	9	0.440	0.594
4	Dadachanji	2015	Asian	rs854560	482	326	808	**80**	353	119	10	215	105	6	0.179	0.091
5	Wang *	2012	Asian	rs854560	610	503	1,113	**91**	570	42	1	473	32	1	0.034	0.557
6	Zhang *	2015	Asian	rs854560	d	d	d	----	429	28	1	409	34	1	0.040	0.742
7	Paltoglu	2013	Caucasian	rs662	142	112	254	35	45	70	27	92	19	1	0.094	0.986
8	San Millan	2004	Caucasian	rs662	d	d	d	----	42	24	6	24	12	6	0.286	0.052
9	Dadachanji	2015	Asian	rs662	d	d	d	----	168	227	87	130	151	45	0.370	0.913
10	Wang	2012	Asian	rs662	d	d	d	----	68	270	272	72	248	183	0.610	0.412
11	Zhang	2015	Asian	rs662	455	441	896	**85**	50	204	201	64	222	155	0.603	0.279
	**LD2**															
1	Ferk	2014	Caucasian	rs705379	118	108	226	32	32	57	29	37	53	18	0.412	0.894
2	Mohammed	2009	African	rs705379	d	d	d	----	22	35	37	29	25	6	0.308	0.961
3	Paltoglu	2013	Caucasian	rs705379	d	d	d	----	40	71	31	36	55	21	0.433	0.999
4	San Millan	2004	Caucasian	rs705379	d	d	d	----	20	26	26	12	26	4	0.405	0.065
5	Zhang (Ch)	2011	Asian	rs705379	346	315	661	73	98	179	69	102	155	58	0.430	0.947
6	Zhang	2015	Asian	rs705379	d	d	d	----	132	229	94	134	233	74	0.432	0.108
7	Dadachanji	2018	Asian	rs705379	516	424	940	**86**	194	247	75	144	199	81	0.426	0.409
8	Liu *PON2*	2019	Asian	rs7493	932	745	1,677	**98**	614	287	31	503	223	19	0.175	0.328
9	Dadachanji	2018	Asian	rs854572 ‡	d	d	d	----	206	233	77	144	194	86	0.432	0.165
	**LD3**															
1	Dadachanji	2018	Asian	rs854573	d	d	d	----	272	192	52	219	165	40	0.289	0.276
2	Dadachanji	2018	Asian	rs705381	d	d	d	----	275	190	51	228	157	39	0.277	0.119
3	Dadachanji	2018	Asian	rs854571	d	d	d	----	199	223	94	164	201	59	0.376	0.836
4	Dadachanji	2018	Asian	rs854572 ‡	d	d	d	----	206	233	77	144	194	86	0.432	0.165

LD: linkage disequilibrium; *: Laplace-corrected genotype data; (Ch) Chinese language; d: duplicate; †: $\alpha = 0.05$; OR 1.50; values in bold under the power column indicate statistically powered studies (> 80%); ‡ LD1-LD2; *wt-wt*: homozygous wild-type genotype; *wt-var*: heterozygous genotype; *var-var*: homozygous variant genotype; HWE: Hardy-Weinberg Equilibrium (HWE numbers indicate P-values)

Table S2 Preferred Reporting Items for Systematic Reviews and Meta-Analyses checklist

Section/topic	#	Checklist item	Reported on page #
TITLE			
Title	1	Identify the report as a systematic review, meta-analysis, or both.	1
ABSTRACT			
Structured summary	2	Provide a structured summary including, as applicable: background; objectives; data sources; study eligibility criteria, participants, and interventions; study appraisal and synthesis methods; results; limitations; conclusions and implications of key findings; systematic review registration number.	2
INTRODUCTION			
Rationale	3	Describe the rationale for the review in the context of what is already known.	3-4
Objectives	4	Provide an explicit statement of questions being addressed with reference to participants, interventions, comparisons, outcomes, and study design (PICOS).	3-4
METHODS			
Protocol and registration	5	Indicate if a review protocol exists, if and where it can be accessed (e.g., Web address), and, if available, provide registration information including registration number.	NA
Eligibility criteria	6	Specify study characteristics (e.g., PICOS, length of follow-up) and report characteristics (e.g., years considered, language, publication status) used as criteria for eligibility, giving rationale.	4
Information sources	7	Describe all information sources (e.g., databases with dates of coverage, contact with study authors to identify additional studies) in the search and date last searched.	4
Search	8	Present full electronic search strategy for at least one database, including any limits used, such that it could be repeated.	4
Study selection	9	State the process for selecting studies (i.e., screening, eligibility, included in systematic review, and, if applicable, included in the meta-analysis).	4
Data collection process	10	Describe method of data extraction from reports (e.g., piloted forms, independently, in duplicate) and any processes for obtaining and confirming data from investigators.	4
Data items	11	List and define all variables for which data were sought (e.g., PICOS, funding sources) and any assumptions and simplifications made.	5
Risk of bias in individual studies	12	Describe methods used for assessing risk of bias of individual studies (including specification of whether this was done at the study or outcome level), and how this information is to be used in any data synthesis.	NA
Summary measures	13	State the principal summary measures (e.g., risk ratio, difference in means).	5-6
Synthesis of results	14	Describe the methods of handling data and combining results of studies, if done, including measures of consistency (e.g., I^2) for each meta-analysis.	6-7

Risk of bias across studies	15	Specify any assessment of risk of bias that may affect the cumulative evidence (e.g., publication bias, selective reporting within studies).	6-7
Additional analyses	16	Describe methods of additional analyses (e.g., sensitivity or subgroup analyses, meta-regression), if done, indicating which were pre-specified.	6-7
RESULTS			
Study selection	17	Give numbers of studies screened, assessed for eligibility, and included in the review, with reasons for exclusions at each stage, ideally with a flow diagram.	6
Study characteristics	18	For each study, present characteristics for which data were extracted (e.g., study size, PICOS, follow-up period) and provide the citations.	7
Risk of bias within studies	19	Present data on risk of bias of each study and, if available, any outcome level assessment (see item 12).	NA
Results of individual studies	20	For all outcomes considered (benefits or harms), present, for each study: (a) simple summary data for each intervention group (b) effect estimates and confidence intervals, ideally with a forest plot.	7-8
Synthesis of results	21	Present results of each meta-analysis done, including confidence intervals and measures of consistency.	7-8
Risk of bias across studies	22	Present results of any assessment of risk of bias across studies (see Item 15).	8
Additional analysis	23	Give results of additional analyses, if done (e.g., sensitivity or subgroup analyses, meta-regression [see Item 16]).	7-8
DISCUSSION			
Summary of evidence	24	Summarize the main findings including the strength of evidence for each main outcome; consider their relevance to key groups (e.g., healthcare providers, users, and policy makers).	8-10
Limitations	25	Discuss limitations at study and outcome level (e.g., risk of bias), and at review-level (e.g., incomplete retrieval of identified research, reporting bias).	11
Conclusions	26	Provide a general interpretation of the results in the context of other evidence, and implications for future research.	12
FUNDING			
Funding	27	Describe sources of funding for the systematic review and other support (e.g., supply of data); role of funders for the systematic review.	NA

Table S3 Comparisons between meta-analyses that examined the *PON* variants associations with PCOS

	This study	Liao et al [20]	Gu et al [19]	Chen et al [18]	Liu et al [21]
Year	2020	2018	2016	2016	2016
Country	Thailand	China	China	China	China
number of studies (forest plot-based)	12	6	5	5	5
number of *PON* variants	8	3	3	3	3
PON2 polymorphism	Yes	No	No	No	No
**Methods and treatments**					
Linkage disequilibrium	Yes	No	No	No	No
Quality assessment	Clark-Baudouin Scale	Newcastle Ottawa Scale	None	None	None
HWE as inclusion criterion	Yes	Yes	Yes	Yes	No
Genetic modeling	Standard	Standard	Allelic	Standard	Standard
Modifier analysis	HWE, power	None	None	HWE	None
Outlier analysis	Yes	No	No	No	No
Heterogeneity tools	Q, I^2	Q, I^2	Q, I^2	Q, I^2	Q, I^2
Subgroup analysis	Ethnicity	Ethnicity<br>Diagnostic criteria<br>Population Hospital<br>Sample size	Ethnicity	None	None
Sensitivity analysis	Yes	Yes	Yes	No	Yes
Publication bias	Yes	Yes	Yes	Yes	Yes

PON: paraoxonase; PCOS: polycystic ovary syndrome; HWE: Hardy Weinberg Equilibrium; Q: χ^2-based Q test; I^2: measure of variability
